# Assessing counselor competence in working with gender nonconforming clients: A randomized study

**DOI:** 10.1371/journal.pmen.0000670

**Published:** 2026-08-06

**Authors:** Brandi V. Nash, Lena Salpietro, Hanadi Y. Hamadi, Kassie R. Terrell, Nicholas de Villiers

**Affiliations:** 1 Department of Public Health, University of North Florida, Jacksonville, Florida, United States of America; 2 School of Lifespan Development and Educational Sciences, Kent State University, Kent, Ohio, United States of America; 3 Department of Health Administration, University of North Florida, Jacksonville, Florida, United States of America; 4 Department of English, University of North Florida, Jacksonville, Florida, United States of America; Michigan State University, UNITED STATES OF AMERICA

## Abstract

Cultural competence in working with gender nonconforming clients is a core ethical and professional obligation in counseling, yet predictors of counselors’ perceived competence remain insufficiently examined. Guided by the Multicultural and Social Justice Counseling Competencies (MSJCCs), this study examined whether demographic characteristics and prior training experiences predicted self-reported competence in working with gender nonconforming clients. Using a randomized experimental survey design, 83 student and professional counselors were assigned to view images of cisgender men with or without makeup, then completed an adapted 12-item Sexual Intervention Self-Efficacy Scale (SISES). Results indicated no significant difference in perceived competence between groups, suggesting that brief situational exposure to gender nonconforming presentation did not affect self-reported competence. Across the full sample, more hours of diversity training, LGBTQIA + /gender-specific training, identifying as a sexual or gender minority, and holding a master’s degree significantly predicted higher self-reported competence. These findings indicate that sustained, targeted professional development contributes to counselor preparedness for gender-affirming practice more than single-exposure interventions.

Counseling is a professional relationship that empowers individuals, families, groups, and communities to accomplish mental health, wellness, education, and career goals through developmentally and culturally responsive interventions [1,2]. Distinct characteristics of counseling include and emphasis on the therapeutic relationship, client empowerment, multicultural humility and responsiveness, advocacy, and the promotion of wellness across diverse populations [1,2]. Cultural competence is a foundational ethical and professional responsibility in the counseling field. This study examined whether counselors’ demographic characteristics and prior training experiences predict self-reported competence in working with gender nonconforming clients. This competence encompasses awareness, knowledge, skills, and advocacy needed to work effectively with diverse populations [1]. Counselor education programs, licensure boards, and accreditation standards also emphasize the importance of ongoing professional development, continuing education, and advocacy to maintain competence. Continuing Education Units (CEUs) play a critical role in helping counselors stay current on evidence-based practices, ethical standards, and culturally responsive interventions [2]. In recent years, the counseling field has increased focus and training on specific intersectional identities, including race/ethnicity and sexual orientation. This targeted approach has demonstrated increased counselor competence and cultural humility [3] when working with diverse clients. With this, we know that targeted training, exposure, and advocacy initiatives can have a significant impact on the counseling field and client outcomes. However, we also know that change often takes time and that systemic barriers continue to impact minoritized communities, one such community being those who are gender diverse, this includes people who are gender nonconforming. Gender diverse individuals warrant particular attention for several reasons: they experience disproportionately high rates of discrimination, mental health disparities, and barriers to affirming healthcare; counselor training has historically emphasized race and sexual orientation with less focus on gender identity and expression; and the rapidly evolving understanding of gender requires ongoing professional development to provide competent care. To aid in building cultural humility and competence, many educators and advocates are working hard to share knowledge, resources, and skills in working with gender diverse clients [4–6]. The purpose of this study was to explore if counselor demographic characteristics and prior training experiences were associated with increased counselor competence when working with gender nonconforming clients.

## Literature review

### Cultural competence: An ethical and legal mandate

Cultural competence is recognized as a fundamental obligation in counseling, formally established through ethical codes, accreditation requirements, and licensure regulations. According to the American Counseling Association [1], counselors are ethically obligated to develop awareness, knowledge, skills, and behaviors that enable them to engage effectively with clients from diverse cultural backgrounds. This ethical mandate includes ensuring that the counseling relationship and intervention strategies are culturally responsive, that biases are examined, and notes that value-based referrals are prohibited [1]. In parallel, the Council for Accreditation of Counseling and Related Educational Programs (CACREP) established explicit curriculum and program standards aimed at ensuring graduates are culturally competent. The 2024 CACREP Standards require that counselor education programs include social and cultural diversity as a foundational curricular area; one that is woven throughout the program rather than covered in a single course [7].

Similarly, state licensure boards embed cultural competence within their requirements, both in initial licensure and ongoing continuing education regulation. For example, Florida statutes require that counseling programs include coursework in social and cultural foundations [8], whereas other states like Georgia explicitly require applicants to have a knowledge of societal trends, culture, subgroups, diversity, and competency in counseling diverse populations [9]. Additionally, licensure boards also often require CEUs in multicultural or diversity topics to maintain competence post-licensure. Although specific hours and definitions vary by state, this requirement reinforces the idea that cultural competence is not static but must be maintained and deepened over time. Together, ACA, CACREP, and state licensure boards require: (1) counselor self-awareness and examination of one’s cultural identity; (2) acquisition of knowledge about diverse clients’ worldviews, history, and sociocultural context; (3) development of culturally responsive skills in assessment and intervention; and (4) following legal and ethical mandates in working with diverse clients.

### Targeted training and continuing education

CEUs are critical in the counseling profession. They ensure that counselors maintain competence, remain current on best practices, and uphold ethical standards throughout their careers. Research demonstrates that effective continuing education can strengthen practitioner self-efficacy, improve client outcomes, and ensure that interventions remain evidence-based and socially responsive [10]. CEUs also provide structured opportunities for counselors to stay current with evolving ethical codes, diagnostic standards, and culturally responsive practices [2].

Systematic reviews indicate that most training programs explicitly address race and ethnicity, with a substantial portion also focusing on sexual orientation, leading to improved counselor knowledge, attitudes, and skills [3,11]. For example, Chao, Wei [12] reported that targeted training related to color-blindness increases cultural competence among counselors by eliminating racial performance gaps, enhancing racial/ethnic identity development, and reducing color-blind racial attitudes. Similarly, Pachankis, Soulliard [13] provided a strong example for targeted training on sexual orientation; they conducted a randomized controlled trial for an 11-week LGBTQ-affirmative CBT training. The program significantly enhanced providers’ cultural competence by expanding their knowledge of minority stress and increasing both familiarity with and application of affirming clinical skills. With this, the counseling profession has demonstrated advancements in cultural competence through targeted training, exposure, and continuing education. However, in past years, multicultural training has largely remained focused on race, ethnicity, and sexual orientation [3,11]. This limited exposure may result in a lack of understanding of best practices for working with gender diverse clients.

### Enhancing competence with gender diverse clients

The Association for Lesbian, Gay, Bisexual, and Transgender Issues in Counseling (ALGBTIC), now the Society for Sexual, Affectional, Intersex, and Gender Expansive Identities (SAIGE), created competencies for counselors, educators, and supervisors in 2009 to address this deficit. The competencies were created to guide the exploration of attitudes, awareness, knowledge, and skills; and provide a framework for training curriculum that was historically lacking in counselor preparation programs [14,15]. These competencies serve as a benchmark for best practices when working with LGBTQIA+ clients. However, the language is older (last updated in 2013), and limitations exist. Terms like nonbinary, gender-expansive, and genderfluid are limited; therefore, understanding and application of inclusive practices for working with gender diverse and gender nonconforming clients may be limited. For example, the documents lack guidance in working with nonbinary clients, use of appropriate pronouns, and impact of healthcare and political issues on gender diverse clients [14,15]. Implementation of these competencies are also not uniformly applied or taught in counselor preparation programs [11]. Beyond gender identity, gender expression represents a distinct dimension of diversity that warrants attention in counselor preparation, this includes best practices in working with gender nonconforming clients.

Gender expression refers to the external manifestation of gender through behavior, clothing, grooming, and presentation; whereas gender nonconforming denotes gender expression that does not align with societal expectations associated with a person’s gender. Gender diverse is broadly used [16] to encompass both gender identity (transgender, nonbinary, genderqueer) and gender expression (presentation that challenges traditional gender norms). This inclusive definition recognizes that clients who express gender in nontraditional ways, regardless of their gender identity, may benefit from counselors who have developed competence and comfort working across the spectrum of gender diversity. The counseling literature has primarily focused on competence with transgender and nonbinary clients, with less attention on how counselors respond to diverse gender expression, including those who are gender nonconforming. Counselors’ comfort with clients who challenge gender norms, regardless of identity, reflects their broader capacity to provide affirming care [17]. Addressing gender expression and gender nonconformance as a component of cultural competence aligns with the MSJCCs’ emphasis on examining one’s own attitudes and biases toward marginalized presentations [18]. Counselors who hold rigid beliefs about gender-appropriate behavior may inadvertently communicate discomfort or judgment to clients who do not conform to traditional gender norms, potentially compromising the therapeutic alliance.

Historically, training programs and professional development have addressed sexual orientation broadly, with limited focus on gender identity. Emerging evidence shows a marked increase in targeted training programs, continuing education, and advocacy efforts specifically designed to improve counselors’ knowledge, skills, and attitudes regarding gender identity [19,20]. One such example includes Pope, St. Germain-Sehr [20], delivering asynchronous LGBTQ+ affirmative training to improve counseling students’ knowledge, clinical preparedness, and advocacy skills when working with transgender, nonbinary, and gender-expansive clients. Findings indicated that students reported increased cultural humility and a deeper understanding of gender diversity, especially regarding language, minority stress, and respectful clinical practice.

These trainings include expanding beyond the gender binary, strategies for affirming practice, self-reflection, cultural humility, and advocacy, which are essential for establishing strong therapeutic alliances with gender diverse clients [19,21]. Professional organizations and advocacy groups have also contributed to this shift by offering workshops, webinars, and competency guidelines that increase visibility and provide structured opportunities for skill development (e.g., Gay, Lesbian, and Straight Education Network (GLSEN); The Trevor Project; World Professional Association for Transgender Health (WPATH)). Despite these advances, questions remain regarding whether increased exposure to targeted training, awareness initiatives, and advocacy participation translates into measurable improvements in counselor competence. Therefore, this study sought to determine whether professional development efforts, such as targeted training and continuing education, are statistically associated with increased counselor self-reported competence in working with gender nonconforming clients, thereby providing empirical insight into whether the field is demonstrating measurable growth in counselor competence when working with gender diverse clients. To add to what is known (e.g., studies that indicate increased competence immediately post-intervention, [20]), we are also seeking to understand if there is a sustained impact of receiving these trainings and later self-reported competence in working with gender nonconforming clients. It is important to acknowledge that the relationship between training and competence may not be straightforward. Counselors who have received gender-specific training have, in most cases, also received training in other diversity-related areas. This raises the question of whether gender-related training confers competencies distinct from general diversity training, or whether observed associations reflect broader training effects. The current study cannot fully disentangle these contributions, and this overlap is addressed as a study limitation. What the study can examine is whether self-reported hours of gender-specific training predict perceived competence over and above general diversity training hours, which a regression analysis can directly address.

### Conceptual framework

This study is guided by the Multicultural and Social Justice Counseling Competencies [22]. The MSJCCs were selected over alternative frameworks (e.g., the original Multicultural Counseling Competencies or the ALGBTIC Competencies) because they explicitly integrate social justice and advocacy dimensions while addressing intersectionality, making them particularly suited for examining counselor competence with marginalized gender presentations. The MSJCCs [18] **are applied in this study by examining how** counselors’ awareness, knowledge, and skills related to gender nonconforming clients are influenced by prior targeted training and continuing education, reflecting the model’s emphasis on culturally responsive and justice-oriented counseling practice. This framework offers a theoretical lens for understanding how targeted professional development may enhance measurable counselor competence with gender nonconforming clients.

The MSJCCs organize competencies across four domains: counselor self-awareness, client worldview, counseling relationship, and counseling and advocacy interventions. Within each domain, competencies address attitudes and beliefs, knowledge, skills, and action. This framework is particularly relevant for understanding counselor competence with gender nonconforming clients because it emphasizes both internal dispositions (awareness of one’s own biases) and external capabilities (knowledge and skills to work effectively across difference).

The MSJCCs also highlight that competence is not static but develops through sustained education, training, and self-reflection. This perspective supports our examination of how accumulated professional development, including hours of diversity and gender-specific training, predicts self-reported competence. By framing competence as a developmental process shaped by both formal education and personal characteristics, the MSJCCs provide a theoretical foundation for understanding why some counselors may report greater confidence in working with gender nonconforming clients than others.

### The present study

The literature demonstrates that cultural competence is a core ethical and professional obligation in counseling, reinforced through accreditation standards, licensure requirements, and continuing education [1,2,7]. Targeted training, CEUs, and advocacy efforts have advanced counselors’ knowledge, skills, and attitudes, particularly with regard to race, ethnicity, sexual orientation, and, more recently, gender identity [3,10,12]. However, most research on counselor competence with gender diverse populations has focused on transgender and nonbinary clients, with limited attention to how counselors perceive and respond to diverse gender expression among cisgender individuals.

The current study addresses this gap by examining whether counselors’ demographic characteristics and professional training experiences predict their perceived competence in working with clients who challenge traditional gender norms. To explore this question, we used an experimental design that presented participants with images of theoretical clients: cisgender men wearing makeup (treatment group) or cisgender men without makeup (control group). This approach was intentionally selected to isolate the effect of gender expression, apart from gender identity, on counselor perceptions. Cisgender men who wear makeup represent a visible challenge to traditional masculinity norms without requiring participants to make assumptions about transgender or nonbinary identity. By using this stimulus, the study examined whether counselors’ self-reported competence was influenced by exposure to nontraditional gender expression, extending beyond the existing literature’s focus on transgender and nonbinary populations. We examined whether participant characteristics, including hours of diversity and gender-specific training, sexual or gender minority identity, and educational attainment predicted self-reported competence across the full sample. These analyses tested whether the field is demonstrating measurable growth in counselor competence when working with clients who present outside traditional gender norms and whether sustained professional development contributes to this competence.

## Materials and methods

### Ethics statement

This study was reviewed and approved by the University of North Florida Institutional Review Board (IRB Approval #: 1573439–1). All participants were 18 years of age or older. Participants provided written informed consent prior to completing the survey. No participants under the age of 18 were enrolled in this study; parental/guardian consent was therefore not required.

### Research design

This study utilized a randomized experimental research survey design. To participate in the study, participants had to be 18 years of age or older, live in the U.S., and be either a counselor-in-training or a professional counselor. In this experiment, participants were randomized into one of two groups: the treatment group or the control group. When completing the survey questionnaire, participants randomized into the treatment group responded to the survey questions after viewing images of cisgender men wearing makeup; the control group received images of cisgender men without makeup and completed the same questionnaire as the treatment group.

The use of static images was selected for two reasons. First, it allowed the study to isolate the effect of gender expression, specifically gender-nonconforming presentation, from gender identity, examining counselor responses to a population underrepresented in the literature. Second, static images provided a standardized, feasible stimulus for an online survey format. Images were selected from a publicly available platform and standardized to present men of similar approximate age and neutral expression, differing only in the presence or absence of makeup. No identity label or backstory was provided with the images. Participants were not told that the pictured individual identified as cisgender; the images were unlabeled so that responses reflected reactions to gender expression rather than identity category. The study’s primary hypothesis was that counselors in the treatment group would report lower perceived competence than those in the control group, reflecting potential discomfort or unfamiliarity with gender-nonconforming expression. Secondary hypotheses posited that more hours of diversity training, LGBTQIA + /gender-specific training, sexual or gender minority identity, and higher educational attainment would predict higher self-reported competence across the full sample.

### Procedures

After Institutional Review Board approval, prospective participants were invited to participate in the study in several ways. The researchers contacted several counseling community agencies, organizations, and universities via email and listservs. If prospective participants met the requirements of the study and chose to participate, they were instructed to select the link in the recruitment email that automatically redirected them to a Qualtrics survey. The Qualtrics survey began with an informed consent document. If the prospective participants provided informed consent to participate in the study, they advanced to the questionnaire that was composed of 33 questions used to gather data regarding each participant’s demographic information, diversity training, and perceived competence in working with the theoretical clients who were pictured. Data were collected between March 27, 2020, and June 1, 2020, and participants provided written consent to participate in the study.

### Measures

#### Demographic and diversity training information.

The demographic questionnaire included age, gender, ethnicity, sexual orientation, level of education, professional status, graduation date, geographical region, and questions regarding diversity training (e.g., general diversity training; training specific to the LGBTQIA+ communities; training related to gender, gender identity, and/or gender expression).

#### Sexual intervention self-efficacy scale (SISES)*.*

Perceived counselor competence was measured using the Sexual Intervention Self-Efficacy Scale (SISES) [23]. The SISES is a 19-item scale with three subscales: Comfort/Bias Self-Efficacy, Skill Self-Efficacy, and Information Self-Efficacy. The instrument has been previously tested for reliability and validity and has “moderate to high internal consistency with clinical psychology graduate students as well as evidence for the scale’s validity” [24]. There were high levels of internal consistency with the sample in the three conceptual subscales: Sex Therapy Skills α = .88, Relaying Sexual Information α = .82, Sexual Comfort/Bias α = .64, Total Sexual Intervention Self-Efficacy Scale α = .92 [24]. For this study, two of the three scales were utilized; the two scales included: Comfort/Bias Self-Efficacy and Skill Self-Efficacy. The Information Self-Efficacy subscale was excluded because its items pertained to conveying medical or sexual information not relevant to the competence construct in this study. The 19-item scale became 12 items with the adjustments for this study. The SISES was created to measure perceived counselor competence with a specific area of expertise (sexual intervention); because there are limited scales available to measure intervention related to gender diverse clients, the scale was adapted to fit the purpose of this study [25]. The adaptation involved substituting the original measure’s presenting problem focus (sexual issues) with the population of interest (gender nonconforming individuals). Specifically, all item references to sexual concerns or sexual behavior were replaced with language referencing gender diversity and gender-nonconforming presentation. For example, an item originally reading “I have the skills to address a client’s sexual concerns” was adapted to read “I have the skills to address a client’s concerns related to gender diversity.” Item wording adaptations followed established guidance for questionnaire adaptation [25]. Responses were reported using a Likert scale ranging from 6 (*strongly disagree*) to 1 (*strongly agree*). Internal consistency for the adapted 12-item scale in the current sample was acceptable (Cronbach’s α = .89).

#### Pictures of theoretical clients.

When prompted to answer the 12-item SISES, participants were randomized into either the control group (wherein they responded to the SISES after viewing pictures of cisgender men without makeup) or the treatment group (wherein they responded to the SISES after viewing cisgender men wearing makeup). Images were reviewed by two members of the research team for comparability on perceived age and expression before inclusion. Both images depicted men with a similar neutral facial expression and were cropped to a head-and-shoulders format. No formal manipulation check was administered to confirm that participants attended to or processed the images; this is acknowledged as a study limitation. The adapted 12-item SISES demonstrated acceptable internal consistency, consistent with the original instrument’s reliability estimates. A formal confirmatory factor analysis was not conducted, given the targeted item reduction and the conceptual similarity between the original and adapted constructs; this is also noted as a limitation.

### Data analyses

Descriptive statistics were calculated for continuous variables and categorical variables for the total sample and by group. Competency statement responses were also summarized. Independent samples t-tests and chi-square tests compared groups on continuous and categorical variables, respectively. Linear regression models tested interaction effects between treatment group and participant characteristics on competency scores. A multiple regression identified significant predictors of competency while controlling for treatment. Sensitivity analyses stratified the sample to examine diversity training and LGBTQIA + /gender training effects separately by group, with and without covariate adjustment. Stata MP17 was used for all data cleaning and analysis.

## Results

Participants included 83 counselors (37 control, 46 treatment) who completed the online survey. The unequal group sizes resulted from the Qualtrics randomization algorithm, which assigns participants in real-time without balancing; this minor imbalance does not substantively affect the validity of group comparisons. The control group viewed an image of cisgender men without makeup, while the treatment group viewed an image of cisgender men with makeup. The data in Table 1 compares various characteristics between a control group (*N* = 37) and a treatment group (*N* = 46) with a total sample size of 83. The mean age of participants was 39.82 years (SD = 13.32), with the control group being slightly older (M = 40.03, SD = 14.40) compared to the treatment group (M = 39.65, SD = 12.55). Most participants in both groups graduated around 2012–2013, with an average of about 11–12 years since graduation (control: M = 11.32, SD = 11.06; treatment: M = 11.67, SD = 9.25; the minimum value of -1 indicates participants who had not yet graduated at the time of data collection). The mean number of hours of diversity training is 15.13 (SD = 22.59), with the control group reporting slightly more hours (M = 15.95, SD = 25.75) than the treatment group (M = 14.48, SD = 19.97). Similarly, the average hours of LGBTQIA+ specific training were 6.97 (SD = 11.87), with the control group reporting 6.61 hours (SD = 9.55) and the treatment group reporting 7.26 hours (SD = 13.55). The counselor competence scores were comparable, with an overall mean of 63.28 (SD = 7.45), and the control group (M = 63.81, SD = 6.42) scored slightly higher than the treatment group (M = 62.85, SD = 8.23).

**Table 1:** Comparison of participant continuous variables between control group (N = 37) and treatment group (N = 46).

		mean	SD	Min	Max	N
Control (cisgender men not wearing makeup)	Age	40.03	14.40	23	71	37
N = 37	Years Since Graduating	11.32	11.06	-1	47	37
	Year Graduated	2013	11.06	1977	2025	37
	Number of hours of diversity training	15.95	25.75	0	100	37
	Number of hours of specific training that included LGBTQIA+ community, gender expression, gender identity, and/or types of gender	6.61	9.55	0	40	37
	Counselor Competence	63.81	6.42	47	72	37
Treatment (cisgender men wearing makeup)	Age	39.65	12.55	23	73	46
N = 46	Years Since Graduating	11.67	9.25	0	38	46
	Year Graduated	2012	9.25	1986	2024	46
	Number of hours of diversity training	14.48	19.97	0	100	46
	Number of hours of specific training that included LGBTQIA+ community, gender expression, gender identity, and/or types of gender	7.26	13.55	0	80	46
	Counselor Competence	62.85	8.23	45	72	46
Total, N = 83	Age	39.82	13.32	23	73	83
	Years Since Graduating	11.52	10.03	-1	47	83
	Year Graduated	2012	10.03	1977	2025	83
	Number of hours of diversity training	15.13	22.59	0	100	83
	Number of hours of specific training that included LGBTQIA+ community, gender expression, gender identity, and/or types of gender	6.97	11.87	0	80	83
	Counselor Competence	63.28	7.45	45	72	83

Most participants were from Florida (55.4%, n = 46), with the South region having the highest representation overall (77.1%, n = 64). The control group had 73% (n = 27) from the South, while the treatment group had 80.4% (n = 37) (Table 2). In terms of gender and sexual orientation, the majority of participants identified as women (78.3%, n = 65), with 73% (n = 27) in the control group and 82.6% (n = 38) in the treatment group. Approximately 23% of participants identified as a sexual or gender minority (24.3%, n = 9 in the control; 21.7%, n = 10 in the treatment). Most participants identified as heterosexual or straight (79.52%, n = 66), with 75.68% (n = 28) in the control group and 82.61% (n = 38) in the treatment group. The sample was predominantly Caucasian or White (80.72%, n = 67), with 86.49% (n = 32) in the control group and 76.09% (n = 35) in the treatment group. The treatment group had a higher percentage of Latino/a/x or Hispanic participants (15.22%, n = 7) compared to the control group (2.70%, n = 1).

**Table 2:** Comparison of participant categorical variables between control group (*N* = 37) and treatment group (*N* = 46).

	Control (Viewed cisgender men not wearing makeup) n = 37		Treatment (Viewed cisgender men wearing makeup) n = 46		Total	
	%	No.	%	No.	%	No.
**Region**						
Northeast	2.7	1	6.5	3	4.8	4
Midwest	16.2	6	8.7	4	12	10
South	73	27	80.4	37	77.1	64
West	8.1	3	4.3	2	6	5
**Gender**						
Women	73	27	82.6	38	78.3	65
Men	21.6	8	13	6	16.9	14
Gender Diverse	5.4	2	4.3	2	4.8	4
**Sexual and gender minority**						
No	75.7	28	78.3	36	77.1	64
Yes	24.3	9	21.7	10	22.9	19
**Sexual orientation**						
Asexual	2.70	1	2.17	1	2.41	2
Bisexual or bicurious	8.11	3	6.52	3	7.23	6
Gay	5.41	2	4.35	2	4.82	4
Heterosexual or straight	75.68	28	82.61	38	79.52	66
Lesbian	5.41	2	4.35	2	4.82	4
Pansexual	2.70	1	2.17	1	2.41	2
Queer	5.41	2	4.35	2	4.82	4
Questioning	2.70	1	2.17	1	2.41	2
Demisexual	2.70	1	2.17	1	2.41	2
**Ethnicity and race**						
Caucasian or White	86.49	32	76.09	35	80.72	67
African American or Black	2.70	1	8.70	4	6.02	5
Latino/a/x or Hispanic	2.70	1	15.22	7	9.64	8
Asian	5.41	2	4.35	2	4.82	4
Prefer not to say	2.70	1	2.17	1	2.41	2
**Level of education**						
Currently completing MS or MA in counseling	13.5	5	8.7	4	10.8	9
MS, MA, ED.S in counseling	62.2	23	65.2	30	63.9	53
PhD, EdD, PsyD in counseling or related field	24.3	9	26.1	12	25.3	21
**Status as a counseling professional**						
Master student in counseling	13.5	5	8.7	4	10.8	9
Postdoctoral student in counseling	8.1	3	6.5	3	7.2	6
Registered mental health counseling intern/associate counselor/seeking licensure	24.3	9	28.3	13	26.5	22
Licensed professional counselor (or state equivalent)	54.1	20	56.5	26	55.4	46
**Did you take a class in diverse populations/multicultural counseling in your counseling program**						
Yes	91.9	34	89.1	41	90.4	75
No	8.1	3	10.9	5	9.6	8
**If you have taken a class in diverse populations/multicultural counseling, did learning objectives include gender identity, gender expression, and/or types of gender?**						
Yes	70.6	24	78	32	74.7	56
No	29.4	10	22	9	25.3	19

Regarding education and professional status, most participants had a master’s degree in counseling (63.9%, n = 53), with 62.2% (n = 23) in the control group and 65.2% (n = 30) in the treatment group. Over half of the participants were licensed professional counselors (55.4%, n = 46), with 54.1% (n = 20) in the control group and 56.5% (n = 26) in the treatment group. Regarding prior training experiences, 90.4% (n = 75) of participants reported completing a course in diverse populations or multicultural counseling during their counseling program, with similar proportions in the control (91.9%, n = 34) and treatment (89.1%, n = 41) groups. Among those who completed such a course, approximately three-quarters (74.7%, n = 56) indicated that the course included learning objectives addressing gender identity, gender expression, and/or types of gender. The treatment group reported slightly higher exposure to gender-related content in their multicultural coursework (78%, n = 32) compared to the control group (70.6%, n = 24), though both groups demonstrated substantial prior educational exposure to these topics.

These findings indicate that the majority of participants had received formal multicultural education during their counseling programs, and that most of this coursework included content related to gender diversity. The slightly higher proportion of treatment group participants who reported gender-related learning objectives (78% vs. 70.6%) was not statistically significant, suggesting comparable prior educational exposure across both experimental conditions. This baseline similarity in prior training supports the validity of comparisons between groups on perceived competence outcomes.

When counselors were asked about their knowledge and techniques, most counselors disagreed that they had very little knowledge of interventions (80.7%, n = 67) or were unfamiliar with techniques (75.9%, n = 63) to treat the client’s presenting problems (Table 3). The majority agreed they knew techniques (77.1%, n = 64) and could teach skills (79.5%, n = 66) to help the client. Regarding comfort and biases, 90.3% (n = 75) disagreed that there were issues they would feel uncomfortable discussing with the client, and 85.5% (n = 71) agreed their biases would not hinder their ability to effectively treat the client.

**Table 3:** Participants responses to competency statements.

	Control (Viewed cisgender men not wearing makeup) n = 37		Treatment (Viewed cisgender men wearing makeup) n = 46		Total	
	%	No.	%	No.	%	No.
**I have very little knowledge of the interventions used to treat the presenting problems of the individual depicted in the picture.**						
Strongly Disagree	29.7	11	39.1	18	34.9	29
Disagree	56.8	21	37	17	45.8	38
Slightly Disagree	8.1	3	13	6	10.8	9
Slightly Agree	5.4	2	10.9	5	8.4	7
**There are issues related to the potential presenting problem that I would not feel comfortable discussing with the theoretical client depicted in the picture.**						
Strongly Disagree	59.5	22	52.2	24	55.4	46
Disagree	32.4	12	37	17	34.9	29
Slightly Disagree	5.4	2	4.3	2	4.8	4
Slightly Agree	2.7	1	6.5	3	4.8	4
**I am unfamiliar with the techniques used to intervene with the individual depicted in the image.**						
Strongly Disagree	40.5	15	37	17	38.6	32
Disagree	37.8	14	37	17	37.3	31
Slightly Disagree	13.5	5	13	6	13.3	11
Slightly Agree	5.4	2	8.7	4	7.2	6
Agree	0	0	2.2	1	1.2	1
Strongly Agree	2.7	1	2.2	1	2.4	2
**If this individual told me they were having problems, I would refer them to another clinician.**						
Strongly Disagree	70.3	26	65.2	30	67.5	56
Disagree	27	10	13	6	19.3	16
Slightly Disagree	0	0	15.2	7	8.4	7
Slightly Agree	2.7	1	4.3	2	3.6	3
Agree	0	0	2.2	1	1.2	1
**I am fairly certain that my own biases will not hinder my ability to effectively treat the individual depicted in the image.**						
Strongly Disagree	5.4	2	8.7	4	7.2	6
Disagree	5.4	2	6.5	3	6	5
Slightly Disagree	2.7	1	0	0	1.2	1
Slightly Agree	5.4	2	6.5	3	6	5
Agree	40.5	15	34.8	16	37.3	31
Strongly Agree	40.5	15	43.5	20	42.2	35
**I know some techniques that can help the individual depicted in the image.**						
Strongly Disagree	0	0	2.2	1	1.2	1
Disagree	2.7	1	4.3	2	3.6	3
Slightly Disagree	0	0	4.3	2	2.4	2
Slightly Agree	18.9	7	13	6	15.7	13
Agree	45.9	17	37	17	41	34
Strongly Agree	32.4	12	39.1	18	36.1	30
**I am able to teach this client specific skills to deal with their presenting problems.**						
Strongly Disagree	0	0	4.3	2	2.4	2
Disagree	2.7	1	4.3	2	3.6	3
Slightly Disagree	2.7	1	4.3	2	3.6	3
Slightly Agree	13.5	5	8.7	4	10.8	9
Agree	54.1	20	37	17	44.6	37
Strongly Agree	27	10	41.3	19	34.9	29
**I think that it would be best to refer this client if they had a concern/problem.**						
Strongly Disagree	54.1	20	65.2	30	60.2	50
Disagree	37.8	14	15.2	7	25.3	21
Slightly Disagree	8.1	3	10.9	5	9.6	8
Slightly Agree	0	0	6.5	3	3.6	3
Agree	0	0	2.2	1	1.2	1
**I will be able to treat this client’s problems even when I don’t necessarily agree with their decisions/actions.**						
Disagree	2.7	1	2.2	1	2.4	2
Slightly Agree	5.4	2	4.3	2	4.8	4
Agree	40.5	15	30.4	14	34.9	29
Strongly Agree	51.4	19	63	29	57.8	48
**This individual is someone that I do not know how to treat.**						
Strongly Disagree	56.8	21	56.5	26	56.6	47
Disagree	35.1	13	30.4	14	32.5	27
Slightly Disagree	5.4	2	6.5	3	6	5
Slightly Agree	2.7	1	4.3	2	3.6	3
Agree	0	0	2.2	1	1.2	1
**I worry that I would seem uncomfortable if this client talked to me about their problems.**						
Strongly Disagree	67.6	25	67.4	31	67.5	56
Disagree	27	10	21.7	10	24.1	20
Slightly Disagree	5.4	2	6.5	3	6	5
Slightly Agree	0	0	4.3	2	2.4	2
**I would probably do more harm than good if I tried to work with the individual depicted in the picture.**						
Strongly Disagree	73	27	76.1	35	74.7	62
Disagree	24.3	9	21.7	10	22.9	19
Slightly Disagree	0	0	2.2	1	1.2	1
Strongly Agree	2.7	1	0	0	1.2	1

Most counselors (86.8%, n = 72) disagreed that they would refer the client to another clinician if told they were having problems, and 85.5% (n = 71) disagreed that it would be best to refer the client if they had a concern/problem. A high percentage (92.7%, n = 77) agreed they could treat the client’s problems even if they didn’t agree with their decisions/actions. In terms of perceived ability, 89.1% (n = 74) disagreed that the client was someone they did not know how to treat, 91.6% (n = 76) disagreed they would worry about seeming uncomfortable if the client talked about their problems, and 97.6% (n = 81) disagreed they would do more harm than good if they tried to work with the client. In summary, the control and treatment groups showed similar response patterns across the competency statements, suggesting comparable perceived competence in treating the theoretical client, despite some minor percentage differences between the groups.

The t-test results in Table 4 show no significant differences between the control and treatment groups on any continuous participant characteristics (all p > 0.05). Effect sizes (Cohen’s d) were very small, ranging from -0.05 to 0.13, indicating negligible group differences. The two groups had similar mean scores on counselor competence, age, years since graduating, hours of diversity training, and hours of specific LGBTQIA + /gender diverse training. These results suggest the groups were highly comparable.

**Table 4:** Differences between treatment and control group on participants continuous characteristics.

Continuous participant characteristics	Control		Treatment				
	M	SD	M	SD	t	p	Cohen’s d
Counselor Competence	63.81	6.42	62.85	8.23	0.58	0.56	0.13
Age	40.03	14.40	39.65	12.55	0.13	0.90	0.03
Years Since Graduating	11.32	11.06	11.67	9.25	-0.16	0.88	-0.03
Number of hours of diversity training	15.95	25.75	14.48	19.97	0.29	0.77	0.06
Number of hours of specific training^a^	6.61	9.55	7.26	13.55	-0.25	0.81	-0.05

Note: ^a^specific training included materials covering LGBTQIA+ community, gender expression, gender identity, and/or types of gender

Table 5 presents interaction terms comparing the treatment effect on counselor competence scores across participant characteristics. The results show no significant differences between the control and treatment groups for any characteristic (all p > 0.05). Interactions were tested for sexual/gender minority status (b = 0.69 to 4.36, SE = 1.84 to 2.79, p = 0.108 to 0.805), education level (b = -3.85 to 4.97, SE = 3.55 to 4.94, p = 0.175 to 0.901), professional status (b = -6.47 to 3.51, SE = 3.60 to 5.38, p = 0.233 to 0.876), taking a multicultural counseling class (b = -6.27 to -2.02, SE = 4.49 to 5.48, p = 0.257 to 0.656), and if that class covered gender diversity (b = -0.29 to 0.35, SE = 2.70 to 3.43, p = 0.901 to 0.974). The non-significant interactions suggest the intervention’s impact was consistent regardless of counselor identities and training backgrounds.

**Table 5:** Difference between treatment and control competency across categorical participant characteristics.

	Coefficient	SE	P
**Treatment X sexual and gender minority (Ref: control, not SGM)**			
Control X SGM	0.69	2.79	0.805
Treatment X not SGM	-2.23	1.84	0.229
Treatment X SMG	4.36	2.68	0.108
**Level of education (Ref: control, currently completing MS or MA in counseling)**			
Treatment X Currently completing MS or MA in counseling	-3.85	4.94	0.438
Control X MS, MA, ED.S in counseling	4.97	3.63	0.175
Treatment X MS, MA, ED.S in counseling	3.20	3.55	0.371
Control X PhD, EdD, PsyD in counseling or related field	0.51	4.10	0.901
Treatment X PhD, EdD, PsyD in counseling or related field	1.90	3.92	0.629
**Status as a counseling professional (control, Master student in counseling)**			
Control X Postdoctoral student in counseling	2.53	5.38	0.639
Control X Registered mental health counseling intern/associate counselor/seeking licensure	0.64	4.11	0.876
Control X Licensed professional counselor (or state equivalent)	3.05	3.68	0.41
Treatment X Master student in counseling	-5.05	4.94	0.31
Treatment X Postdoctoral student in counseling	-6.47	5.38	0.233
Treatment X Registered mental health counseling intern/associate counselor/seeking licensure	3.51	3.88	0.368
Treatment X Licensed professional counselor (or state equivalent)	1.62	3.60	0.653
**Did you take a class in diverse populations/multicultural counseling in your counseling program (Ref: Control, no)**			
Control X Yes	-2.02	4.52	0.656
Treatment X Yes	-2.40	4.49	0.595
Treatment X No	-6.27	5.48	0.257
**If you have taken a class in diverse populations/multicultural counseling, did learning objectives include gender identity, gender expression, and/or types of gender? (Ref: Control, no)**			
Control X Yes	0.35	2.81	0.901
Treatment X Yes	-0.09	2.70	0.974
Treatment X No	-0.29	3.43	0.933
Number of hours of diversity training			
Treatment X Increased training	0.06	0.07	0.404
Number of hours of specific training			
Treatment X Increased specific training	0.03	0.15	0.825

A linear regression was used to predict counselor competency from participant characteristics, controlling for the non-significant treatment effect (Table 6). More hours of diversity training (b = 0.11, p = 0.002) and LGBTQIA + /gender diverse training (b = 0.19, p = 0.007), identifying as a sexual or gender minority vs not (b = 3.84, p = 0.049), and having a master’s degree vs being a current student (b = 5.82, p = 0.031) significantly predicted higher competency scores. Age, years since graduating, being licensed or a registered intern, taking a multicultural counseling class, and that class covering gender were not significant predictors (p > 0.05).

**Table 6:** Linear Regression of participant competency score across participant characteristics controlling for treatment.

	Coefficient	SE	P
Age	0.01	0.06	0.843
Years Since Graduating	-0.09	0.08	0.278
Number of hours of diversity training	0.11	0.03	0.002
Number of hours of specific training that included the LGBTQIA+ community, gender expression, gender identity, and/or types of gender	0.19	0.07	0.007
**Sexual and gender minority (Ref: No)**			
Yes	3.84	1.92	0.049
**Level of education (Ref: Currently completing MS or MA in counseling program)**			
MS, MA, ED.S in counseling	5.82	2.64	0.031
PhD, EdD, PsyD in counseling or related field	3.17	2.92	0.281
**Status as a counseling professional (Ref: Master student in counseling)**			
Postdoctoral student in counseling	0.35	3.91	0.929
Registered mental health counseling intern/associate counselor/seeking licensure	4.77	2.94	0.109
Licensed professional counselor (or state equivalent)	4.64	2.71	0.091
**Did you take a class in diverse populations/multicultural counseling in your counseling program (Ref: no)**			
Yes	1.62	2.80	0.565
**If you have taken a class in diverse populations/multicultural counseling, did learning objectives include gender identity, gender expression, and/or types of gender? (Ref: no)**			
Yes	0.28	1.97	0.889
^a^Controlled for treatment effect which was not significant			

Lastly, a sensitivity analysis of counselor competency in the control and treatment groups is presented in Table 7. In unadjusted models, diversity training hours significantly predicted competency in both groups (control: b = 0.09, p = 0.033; treatment: b = 0.15, p = 0.016). LGBTQIA + /gender diverse training hours were only significant in the treatment group (b = 0.19, p = 0.03). In adjusted models controlling for region, sexual and gender minority status, education, professional status, multicultural class, gender topics, and years since graduating, the effects were no longer significant.

**Table 7:** Sensitivity analysis of counselor competency in both the control and treatment group.

	Control			Treatment		
	Coefficient	SE	P	Coefficient	SE	P
**Unadjusted model**						
Number of hours of diversity training	0.09	0.04	0.033	0.15	0.06	0.016
Number of hours of specific training	0.16	0.11	0.152	0.19	0.09	0.03
**Adjusted model**						
Number of hours of diversity training*	0.09	0.05	0.102	0.12	0.06	0.061
Number of hours of specific training *	0.24	0.13	0.076	0.16	0.09	0.07

*stratified analysis of within treatment and control and controlled for region, GSM, Level of education, Status as a counseling professional, If you have taken a class in diverse populations/multicultural counseling, did learning objectives include gender identity, gender expression, and/or types of gender?, years since graduating.

Note: specific training included the LGBTQIA+ community, gender expression, gender identity, and/or types of gender.

## Discussion

The primary purpose of this study was to examine whether counselors’ professional training experiences and demographic characteristics predicted their perceived competence in working with gender nonconforming clients. Consistent with prior literature suggesting that targeted training and education enhance multicultural counseling competence [3,19], the results revealed no significant difference in perceived competence between counselors who viewed a gender nonconforming client and those who did not. However, across the full sample, greater hours of diversity and gender-specific training, identifying as a sexual or gender minority, and holding a master’s degree were significantly associated with higher self-reported competence. These findings emphasize the continued importance of specialized training and education in fostering counselor competence with gender diverse populations.

### Perceived competence and influence of training

A comparison between the treatment and control groups revealed similar levels of perceived competence, indicating that viewing an image of a gender nonconforming client (i.e., a cisgender man wearing makeup) did not significantly influence counselors’ self-reported competence. This null finding shows that the treatment condition itself did not affect perceived competence. Although direct comparisons are limited, prior literature has emphasized the role of multicultural and gender-focused training in promoting counselor awareness, knowledge, and perceived skill when working with gender-diverse clients [26–28]. The lack of significant differences between the treatment and control groups, along with the absence of significant interaction effects across participant characteristics (e.g., education level, professional status, sexual or gender minority identity, and training exposure), further supports the interpretation that counselors’ self-assessed competence reflects long-term professional development rather than situational factors. Few studies have experimentally examined counselor responses to gender-nonconforming expression, making direct comparisons difficult. Most prior research has focused on evaluating the effects of affirmative training interventions and educational experiences on counselor competence [20,26–28] rather than examining whether exposure to a particular client presentation influences self-reported competence. As such, the present study extends the literature by exploring a previously understudied question regarding gender expression and counselor competence.

While these results could suggest that stigma or bias may not overtly influence counselors’ self-perceptions of competence with gender nonconforming clients, alternative explanations are possible. First, the experimental manipulation may have been too subtle to elicit measurable differences in perceived competence. Static images of cisgender men with or without makeup may not have provided sufficient stimulus intensity to activate biases or discomfort that would influence self-reported competence; more immersive stimuli such as video vignettes or interactive role-plays might yield different results. Second, given the self-report nature of the survey and the professional expectations of unconditional positive regard in counseling and cultural competence [1,7], participants may have been motivated to report high competence regardless of their actual comfort or skill. This interpretation aligns with prior research showing discrepancies between self-perception of competence and external evaluation of competence. For example, research has shown that supervisees are more likely to rate themselves as more competent compared to their supervisors’ ratings of them [29]. Thus, the consistently high perceived competence scores in this study may reflect aspirational self-assessments rather than actual preparedness or comfort in working with gender nonconforming clients. To better understand what factors contributed to counselors’ perceived competence, we next examined predictors across the full sample, controlling for the non-significant treatment effect.

### Predictors of perceived competence

Because the experimental comparison produced no significant group differences, the following analyses shift from the randomized comparison to an examination of predictors of perceived competence across the full sample. This analytic pivot was planned a priori as the study’s secondary aim: to identify counselor-level characteristics associated with competence regardless of experimental condition. Readers should interpret findings from this section within the constraints of a cross-sectional, self-report design; the associations described do not carry causal inference. The survey-based Likert items are the basis for these analyses, and the discussion focuses on what these self-report responses reveal about the relationship between professional development and perceived competence. In these analyses, age, years since graduation, professional status, and completion of a multicultural counseling class that included gender topics were not significant predictors of competence. By contrast, counselors who reported more hours of diversity and gender-related training showed higher self-reported competence in unadjusted models, but these associations were not statistically significant after covariate adjustment, though point estimates remained positive, indicating that greater training hours continued to correspond with slightly higher perceived competence. This pattern suggests that while counselors with more training tend to perceive themselves as more competent, the relationship is not strong enough to reach statistical significance once other factors are considered. This attenuation of effects following adjustment suggests potential confounding, with variables such as education level and professional status likely sharing variance with training hours. Specifically, counselors with more advanced degrees or professional experience may have had more opportunities for training, making it difficult to isolate the unique contribution of training hours. This could indicate that additional influences (e.g., individual attitudes, professional environment, or prior exposure) may also shape perceptions of competence.

These findings are consistent with several previous studies examining counselor competence with LGBTQIA+ populations. LGBTQIA+ ally training has been associated with greater counselor competency [27], and school counselors with greater preparation related to LGBTQIA+ populations have demonstrated higher competence when working with students [28]. Similarly, training and professional development have been identified as important factors associated with competence in working with transgender students [26], and LGBTQ + -affirmative training has been shown to increase counseling students’ preparedness to work with transgender, nonbinary, and gender-expansive clients [20]. Collectively, this suggests that targeted educational experiences may contribute to counselors’ perceptions of preparedness when working with gender-diverse populations.

We also found that, when controlling for the treatment effect, counselors with a master’s degree reported significantly higher perceived competence compared to those currently enrolled in master’s programs. Counselors who identified as a sexual or gender minority also reported higher perceived competence. Although relatively little research has directly examined this relationship among counseling professionals, higher competence among counselors who identified as a sexual or gender minority is broadly consistent with scholarship emphasizing the importance of familiarity with LGBTQIA+ experiences and identities when providing affirming care [26,30]. It is possible that personal experiences related to identity development, minority stress, or engagement with LGBTQIA+ communities contribute to greater confidence when working with gender-diverse clients. However, the present study cannot determine the mechanisms underlying this association. Higher competence among counselors with a master’s degree suggests that perceived competence in working with gender diverse clients may increase as counselors progress through their formal education and acquire more exposure to multicultural and gender-related training. Notably, this finding may also reflect general confidence gained through educational completion rather than specific competence development with gender diverse populations. Although there is limited evidence that professional experience alone, beyond completion of a degree, leads to higher perceived competence, post-graduate professional development and continuing education have been shown to enhance competence [26].

The primary contribution of this study is its extension of the counselor competence literature beyond the transgender and nonbinary focus that has dominated recent scholarship. By using cisgender men wearing makeup as the experimental stimulus, the study explicitly examined counselor responses to gender nonconforming expression among cisgender individuals, which is a population largely absent from the competence literature. To our knowledge, this is among the first studies in the counseling literature to experimentally examine counselor responses to gender nonconforming expression independent of transgender or nonbinary identity. By focusing on gender expression rather than gender identity, the study broadens understanding of how counselor competence may apply across diverse presentations of gender. This design tests whether counselor competence frameworks apply to gender expression diversity, not only gender identity diversity, addressing a gap noted in the literature on ALGBTIC and SAIGE competencies [14,15]. The null experimental finding itself carries theoretical significance: it suggests counselors’ self-perceptions of competence are not destabilized by a single gender-nonconforming image, which may reflect genuine professional confidence, consistent training exposure across the sample, or social desirability. The regression findings add to a small but growing body of evidence that sustained training hours, not isolated exposure, predict perceived preparedness, with direct implications for how counselor education program’s structure continuing education requirements around gender diversity.

### Limitations and recommendations for future research

Several limitations exist in the present study. First, the study relied on volunteer participants, which may have introduced self-selection bias if individuals with greater interest or experience in diversity topics were more likely to participate. Second, because the study used self-report measures, participants may have underreported their biases or overestimated their competence, particularly given the counseling profession’s emphasis on unconditional positive regard, nondiscrimination, and cultural humility [1,7]. Clinicians who internalize these values may report high competence regardless of their actual clinical preparedness. This tendency can create ceiling effects in data. These effects hide the true differences in actual skill levels. This issue is likely due to the sample composition. Most participants were licensed or pre-licensed professionals who completed multicultural training. Therefore, they could easily identify the socially desirable answers on a competence scale. The high scores in both groups support this idea. Prior studies show that self-reported competence often differs from external evaluations (e.g., counselors regularly rate themselves higher than their supervisors do) [29]. Future research should continue examining how educational and professional experiences contribute to both perceived and demonstrated competence. Longitudinal and observational designs could clarify whether increased training and education translate into observable counseling behaviors and client outcomes. Expanding samples to include a broader range of counselors and related professionals (e.g., social workers, psychologists, psychiatrists) would enhance generalizability. Researchers might also refine measurement tools to more accurately assess both self-perceived and observed competence. Collectively, these directions can deepen understanding of how counselors’ training, education, and lived experiences shape their perceived competence to provide affirming care to gender nonconforming individuals.

## Conclusions

This study explored the relationship between counselors’ professional training experiences, demographic characteristics, and self-reported competence in working with gender nonconforming clients. Results indicate that exposure to targeted professional development, including diversity training and LGBTQIA + /gender-specific training, along with identifying as a sexual or gender minority and holding a master’s degree, are associated with higher perceived competence. Conversely, situational exposure to gender nonconforming client images did not significantly impact self-reported competence, suggesting that long-term training and professional experiences play a larger role than brief, isolated interventions.

These findings highlight the importance of sustained, structured, and targeted professional development in fostering cultural competence and humility when working with gender diverse populations. Counselor education programs and professional organizations should continue to expand training opportunities that address gender diversity, including nonbinary and gender-expansive identities, to ensure that all counselors are equipped to provide affirming, culturally responsive care. Overall, the study reinforces the ethical and professional mandate for counselors to engage in ongoing education, self-reflection, and advocacy to meet the evolving needs of gender diverse clients.
